# Understanding variation in person-centered maternity care: Results from a household survey of postpartum women in 6 regions of Ethiopia

**DOI:** 10.1016/j.xagr.2022.100140

**Published:** 2022-12-05

**Authors:** Elizabeth K. Stierman, Linnea A. Zimmerman, Solomon Shiferaw, Assefa Seme, Saifuddin Ahmed, Andreea A. Creanga

**Affiliations:** 1 Department of International Health, Johns Hopkins Bloomberg School of Public Health, Baltimore, MD (Drs Stierman and Creanga); 2 Department of Population, Family, and Reproductive Health, Johns Hopkins Bloomberg School of Public Health, Baltimore, MD (Drs Zimmerman and Ahmed); 3 School of Public Health, Addis Ababa University, Addis Ababa, Ethiopia (Drs Shiferaw and Seme); 4 Department of Gynecology and Obstetrics, Johns Hopkins Medicine, Baltimore, MD (Dr Creanga)

**Keywords:** Ethiopia, healthcare disparities, obstetrics, person-centered maternity care, quality of care, respectful maternity care

## Abstract

**BACKGROUND:**

Effective communication, respect and dignity, and emotional support are critical for a positive childbirth experience that is responsive to the needs and preferences of women.

**OBJECTIVE:**

This study evaluated the performance of a person-centered maternity care scale in a large, representative household sample of postpartum women, and it describes differences in person-centered maternity care across individuals and communities in Ethiopia.

**STUDY DESIGN:**

The study used data from 2019 and 2020 from a representative sample of postpartum women in 6 regions of Ethiopia. It measured person-centered maternity care using a scale previously validated in other settings. To assess the scale validity in Ethiopia, we conducted cognitive interviews, measured internal consistency, and evaluated construct validity. Then, we fit univariable and multivariable linear regression models to test for differences in mean person-centered maternity care scores by individual and community characteristics. Lastly, multilevel modeling separated variance in person-centered maternity care scores within and between communities.

**RESULTS:**

Effective communication and support of women's autonomy scored lowest among person-centered maternity care domains. Of 1575 respondents, 704 (44.7%) were never asked their permission before examinations and most said that providers rarely (n=369; 23.4%) or never (n=633; 40.2%) explained why procedures were done. Person-centered maternity care was significantly higher for women with greater wealth, more formal education, and those aged >20 years. Variation in person-centered maternity care scores between individuals within the same community (τ^2^=58.3) was nearly 3 times greater than variation between communities (σ^2^=21.2).

**CONCLUSION:**

Ethiopian women reported widely varying maternity care experiences, with individuals residing within the same community reporting large differences in how they were treated by providers. Poor patient-provider communication and inadequate support of women's autonomy contributed most to poor person-centered maternity care.

**AJOG Global Reports at a Glance**

**Why was this study conducted?**

Person-centered maternity care (PCMC)—care that is responsive to the needs, preferences, and values of women—is important to uphold trust in the health system and to encourage healthcare-seeking behaviors. This study assessed PCMC in a representative sample of Ethiopian women using a validated scale.

**Key findings**

Facility childbirth experiences varied widely. Individuals with no or limited formal education, lower wealth, and adolescents reported less respectful treatment by healthcare providers than their counterparts.

**What does this add to what is known?**

Efforts to measure differences in PCMC across sub-populations have been hindered by the lack of large-scale, population-based studies. As one of the few, our study provides evidence of systematic sociodemographic differences in PCMC, underscoring the importance of centering equity in quality improvement efforts in health facilities.

## Introduction

An estimated 140 million births occur each year worldwide.^1^ A growing proportion of these births take place in a health facility—76% of births globally according to data from 2015 to 2020.^2^ However, the experience of women delivering in health facilities varies widely and, unfortunately, disrespect and abuse of patients during childbirth is far too common. A study led by the World Health Organization (WHO) in 4 low- and middle-income countries (LMICs) found that more than one-third of women delivering in health facilities experienced mistreatment; this included physical and verbal abuse, stigma and discrimination, and failures to uphold professional standards (eg, nonconsented procedures, neglect).^3^ In 2014, the WHO issued a statement calling for the prevention and elimination of disrespect and abuse during facility-based childbirth,^4^ and in 2015, the WHO published a vision for quality maternal and newborn care that is safe, effective, timely, efficient, equitable, and person-centered.^5^ This framework broadened the discussion on maternity care experiences to include not only the absence of mistreatment but also its positive corollaries, namely effective communication, respect and dignity, and emotional support. These domains are critical for a positive childbirth experience that is responsive to the needs and preferences of women and, over the long-term, enhances trust in the health system and encourages healthcare-seeking behaviors.^6^, ^7^, ^8^, ^9^, ^10^, ^11^, ^12^, ^13^, ^14^, ^15^

Maternity care experiences are influenced by many factors, including patients’ expectations, providers’ beliefs and biases, and a range of contextual variables related to the health system and the social environment.^14^, ^15^, ^16^, ^17^, ^18^, ^19^, ^20^, ^21^, ^22^, ^23^, ^24^, ^25^ These latter contextual factors include health system management, financing, infrastructure, and policies, as well as community norms and population characteristics. Understanding these factors is key to effectively design and target respectful maternity care interventions. Although data exist on how treatment varies by patient and provider characteristics,^13^^,^^26^, ^27^, ^28^, ^29^, ^30^, ^31^, ^32^, ^33^, ^34^, ^35^, ^36^, ^37^, ^38^, ^39^, ^40^ limited data are available on the influence of contextual factors on maternity care experiences in Ethiopia.^16^^,^^19^ To our knowledge, no studies have examined the extent to which person-centered maternity care (PCMC) varies across and within communities. This is important for understanding how much maternity care experiences depend on individual-level interactions between providers and patients relative to the broader environment in which these interactions take place.

This study sought to assess and compare maternity care experiences of a representative sample of women residing in 6 regions of Ethiopia that together represent 90% of the country's population using a validated scale of PCMC.^41^ Our first objective was to assess the performance of the PCMC scale in Ethiopia and to compare the results with previous validation studies in other countries. Second, we aimed to identify individual and community predictors of PCMC and to explain variations in maternity care experiences across individuals and communities in Ethiopia.

## Materials and Methods

### Study design and procedures

This study used data collected between September 2019 and September 2020 from a representative sample of postpartum women aged 15 to 49 years across 6 regions in Ethiopia, namely Addis Ababa, Afar, Amhara, Oromia, the Southern Nations, Nationalities, and Peoples’ Region (SNNPR), and Tigray. (Note: SNNPR was subsequently divided into three regions: SNNPR, Sidama, and South West Ethiopia People's Region (SWER); results are representative of political boundaries in September 2019.) This study followed a multistage sampling procedure as follows: strata were first defined by region and urban or rural designation, and then, enumeration areas (EAs) were randomly selected from each stratum with probability proportional to size. A household census identified eligible women residing in sampled EAs. Women who were pregnant or <6 weeks postpartum were invited to enroll in the study, and those who consented received a baseline survey and follow-up surveys at 6 weeks, 6 months, and 1 year postpartum. In this study, the analysis of the 6 week postpartum survey data for women who delivered at a health facility is shown. In addition, a household survey conducted in the same EAs between September 2019 and December 2019 provides data on community characteristics. The study design and procedures for sampling, questionnaire development, and survey implementation are described in the study protocol, available elsewhere.^42^

The survey was administered as part of Performance Monitoring for Action Ethiopia (PMA-ET), a project implemented by the Addis Ababa University School of Public Health and the Johns Hopkins Bloomberg School of Public Health, and was funded by the Bill & Melinda Gates Foundation (INV 009466). PMA-ET received ethical approval from the Addis Ababa University, College of Health Sciences (reference number AAUMF 01-008) and the Johns Hopkins University Bloomberg School of Public Health Institutional Review Board (FWA00000287).

### Measurement of person-centered maternity care

We used the 13-item short version of the PCMC scale developed by Afulani et al^41^ to measure the experience of women delivering in health facilities (appendix pp. 2-3). This short PCMC scale correlates with a longer 30-item scale validated in Kenya, India, and Ghana.^43^, ^44^, ^45^ Before administering the scale in the PMA-ET study, the postpartum questionnaire was piloted in Addis Ababa and the surrounding Oromia zones in May 2019; cognitive interviews (n=5) assessed women's comprehension of the PCMC items.^42^

Each item in the PCMC scale had 4 possible responses given a numeric value of zero (“no, never”), 1 (“yes, a few times”), 2 (“yes, most of the time”), or 3 (“yes, all the time”). Multivariate imputation using chained equations was applied to impute missing values; one respondent who answered “did not remember” to all 13 questions was excluded from analysis, yielding an analytical sample of 1575 women. After imputation, we added the numeric ratings for each item to produce a summary score, which had a minimum value of zero and a maximum value of 39.

### Statistical analysis

We assessed internal consistency of the PCMC scale using Cronbach's alpha and evaluated construct validity by measuring the association between PCMC and receipt of a maternal postpartum check by a healthcare provider before discharge from the facility—a key maternal health indicator expected to correlate with PCMC.

Next, we assessed differences in the PCMC scores by individual and community characteristics. Individual characteristics included women's age, marital status, religion, education, wealth quintile, receipt of at least 4 antenatal care contacts or visits, whether family and friends were allowed during labor, place of delivery, type of provider attending the delivery, birth outcome, vaginal or cesarean delivery, and whether the respondent self-reported complications during delivery or the first 24 hours postpartum. Community characteristics included rural or urban location, region, percentage of women in the respondent's community with a secondary (or higher) education, percentage of households in the respondent's community that were poor (ie, categorized in the lowest 3 wealth quintiles based on a household asset index), and community norms about facility delivery. Community norms were measured by the question “Do most, some, few, or no people in your community encourage women to deliver at a facility?”. Response options were recoded using a numeric scale as zero (“no people”), 1 (“few people”), 2 (“some people”), and 3 (“most people”), and the community average was calculated. We fit univariable and multivariable linear regression models to test for differences in the mean PCMC scores by subgroups.

We assessed multicollinearity by using variance inflation factors and by comparing regression models with and without specific covariates. Covariates with substantial multicollinearity were removed from multivariable linear regression models. Sensitivity analysis explored the effect of the COVID-19 pandemic on the results by adding a variable to the regression models to examine differences among respondents who delivered before April 8, 2020, when a state of emergency was declared in Ethiopia, and those who delivered on April 8, 2020, or later. Coefficients and standard errors were weighted to account for complex survey design, clustering within EAs, and variability between imputations using the mi estimate: svy commands in Stata (version 15) (StataCorp LLC, College Station, TX).^46^

Finally, we used graphical displays and multilevel modeling to separate the variance in PCMC scores among women residing within the same community from the variance associated with differences between communities. This analytical strategy was adapted from methods for the analysis of contextual phenomena using multilevel models.^47^, ^48^, ^49^ We first fit an empty model with a random intercept for community and no covariates. Next, we fit 3 random-intercept models adjusted for (1) community covariates, (2) individual covariates, and (3) all covariates with the aim of assessing the importance of individual effects relative to community effects in predicting a woman's maternity care experience. Coefficients and standard errors were adjusted for the complex survey design and for variability between imputations using the “mi estimate: mixed” commands in Stata (version 15) with a random intercept for community and sampling weights (“pweight”) to account for the EA selection probability. We compared the random effects of the multilevel models, which quantified the variance between communities (τ^2^) and the variance between individuals within communities (σ^2^). Differences in the deviation of the PCMC scores between urban and rural communities were tested using 1-way analysis of variance.

## Results

### Sample characteristics

Among the 1575 postpartum women in our analytical sample, 938 (59.5%) were between the ages of 20 and 29 years (Table 1). Approximately one-third (n=477; 30.3%) had a secondary education or higher. The primary religions of respondents were Orthodox (n=720; 45.7%), Muslim (n=464; 29.5%), and Protestant (n=368; 23.3%). A total of 607 (38.5%) resided in urban areas, and most were located in Ethiopia's 3 most populous regions, namely Oromia (n=639; 40.6%), Amhara (n=341; 21.7%), and SNNPR (n=327; 20.8%). Most respondents gave birth at a government health center (n=911; 58.0%) or a government hospital (n=594; 37.7%).

**Table 1:** Sample characteristics

Characteristics	Maternity patients (N=1575)	
	Weighted count	Weighted percentage/mean (95% CI)
**Individual characteristics**		
Age group, %		
<20 y	177	11.2 (8.6–13.8)
20–24 y	442	28.0 (24.9–31.1)
25–29 y	496	31.5 (28.7–34.3)
30–34 y	256	16.3 (14.1–18.4)
>35 y	205	13.0 (10.8–15.2)
Currently married,^a^ %	1490/1574	94.6 (93.0–96.3)
Religion		
Protestant	368	23.3 (18.3–28.3)
Orthodox	720	45.7 (39.9–51.5)
Muslim	464	29.5 (21.8–37.1)
Other	24	1.5 (0.4–2.6)
Wealth quintile, %		
Lowest	165	10.5 (7.6–13.3)
Lower	223	14.1 (11.7–16.6)
Middle	285	18.1 (14.9–21.3)
Higher	353	22.4 (18.3–26.5)
Highest	550	34.9 (30.2–39.6)
Education, %		
Never attended	451	28.6 (25.1–32.2)
Primary	647	41.1 (37.4–44.8)
Secondary	275	17.5 (14.7–20.2)
Higher	202	12.8 (10.3–15.3)
Had at least 4 antenatal care visits or contacts,^b^ %	904/1572	57.5 (52.3–62.7)
Had a cesarean delivery, %	159	10.1 (8.3–12.0)
Self-reported complications during delivery, %	689	43.7 (39.6–47.9)
Self-reported complications in first 24 h following delivery, %	461	29.3 (25.1–33.4)
Experienced a stillbirth,^c^ %	29	1.8 (1.0–2.7)
Allowed family and friends during labor, %	780/1570	49.7 (44.9–54.5)
Place of delivery, %		
Government hospital	594	37.7 (32.2–43.1)
Government health center	911	58.0 (52.3–63.4)
Government health post	18	1.2 (−0.4 to 2.7)
Private facility^d^	51	3.3 (2.1–4.4)
Provider attending delivery, %		
Doctor	304	19.3 (16.1–22.5)
Health officer	18	1.1 (0.3–1.9)
Nurse or midwife	699	44.4 (39.0–49.7)
Skilled attendant––cannot distinguish	552	35.0 (29.9–40.1)
Health extension worker	3	0.2 (−0.1 to 0.4)
Community characteristics		
Urban residence, %	607	38.5 (34.4–42.7)
Region, %		
Tigray	145	9.2 (7.7–10.7)
Afar	9	0.6 (0.1–1.1)
Amhara	341	21.7 (18.0–25.3)
Oromia	639	40.6 (35.8–45.3)
SNNPR^e^	327	20.8 (17.2–24.4)
Addis Ababa	114	7.2 (5.8–8.6)
Poor households in community,^f^ mean %	820	52.1 (47.0–57.2)
Women in community with secondary or higher education,^g^ mean %	464	29.5 (26.1–32.8)
Community norms about facility delivery,^h^ mean score	NA	2.53 (2.44–2.62)

Estimates weighted to account for complex survey design. Categories may not sum to 1575 because of rounding.

*CI*, confidence interval; *NA*, not applicable; *SNNPR*, Southern Nations, Nationalities, and Peoples’ Region.

a Currently married or living together as if married

b Antenatal contacts or visits were with any healthcare provider or health extension worker in a facility, home, or other location

c In case of multiple births, categorized as stillbirth if any of the births were stillbirths. This reflects the percentage of women in the sample who experienced a stillbirth (not the stillbirth rate)

d Includes facilities managed by private for-profits, nongovernmental organizations, and faith-based organizations

e SNNPR was subsequently divided into 3 regions, namely SNNPR, Sidama, and South West Ethiopia People's Region (SWER); results are representative of political boundaries in September 2019

f Among surveyed women, the mean percentage of households that were poor (lowest, lower, middle wealth quintiles) in the community where the participant resided

g Among surveyed women, the mean percentage of women with a secondary education or higher in the community where the participant resided

h Among surveyed women, the mean score for norms about facility delivery in the community where the participant resided. The score had a minimum of zero (all women respondents in the community perceived that no people encourage facility delivery) and maximum of 3 (all female respondents in the community perceived that most people encourage facility delivery). *Stierman. Person-centered maternity care in Ethiopia. Am J Obstet Gynecol Glob Rep 2022.*

### Performance of 13-item PCMC scale

All 13 items of the PCMC scale were retained after cognitive testing and the pilot survey. Response categories remained the same as the original categories developed by Afulani et al^41^ except for 1 item, the question on whether women felt they were able to be in the position of their choice during delivery, which was revised to a yes or no response. Response rates to the 13-item PCMC scale were high (appendix p.4), including among women of varying educational levels and in both urban and rural settings. Internal consistency of the scale was high (Cronbach's alpha=0.89), similar to previous validation studies (appendix p.5). Indicative of construct validity, receipt of a maternal postpartum check before discharge from the facility had a significant association with the PCMC scores (*P*<.001). For each unit increase in a woman's PCMC score, the odds of receiving a maternal postpartum check increased by 9% (odds ratio, 1.09; 95% confidence interval [CI], 1.06–1.11).

### Description of maternity care experiences

**Figure 1:**
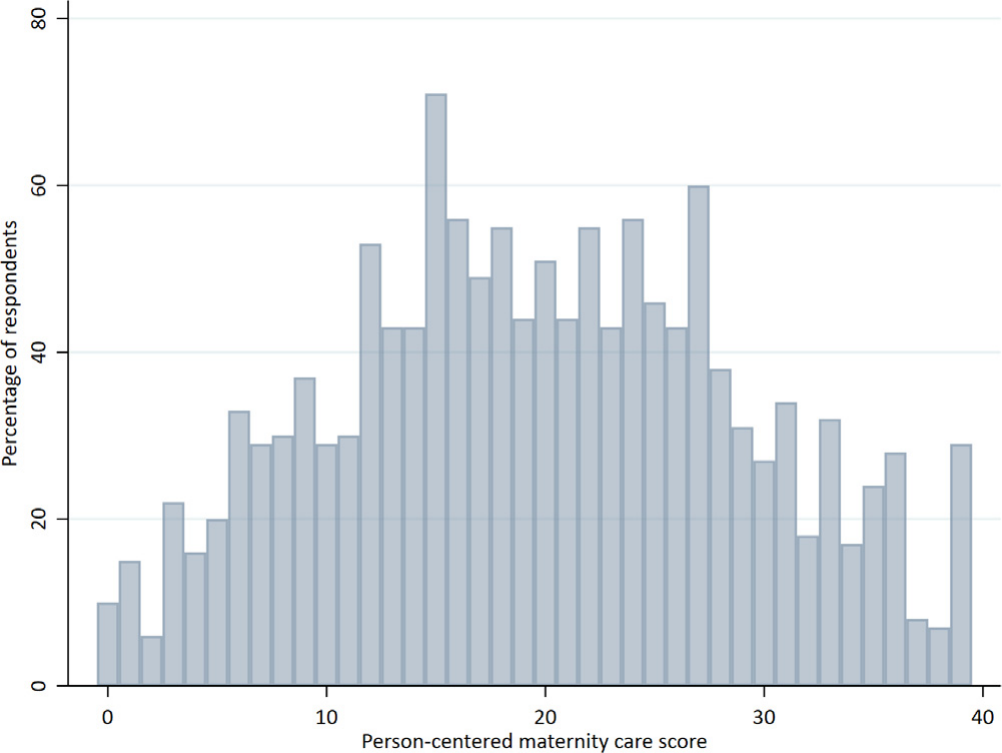
Distribution of person-centered maternity care scores

**Figure 2:**
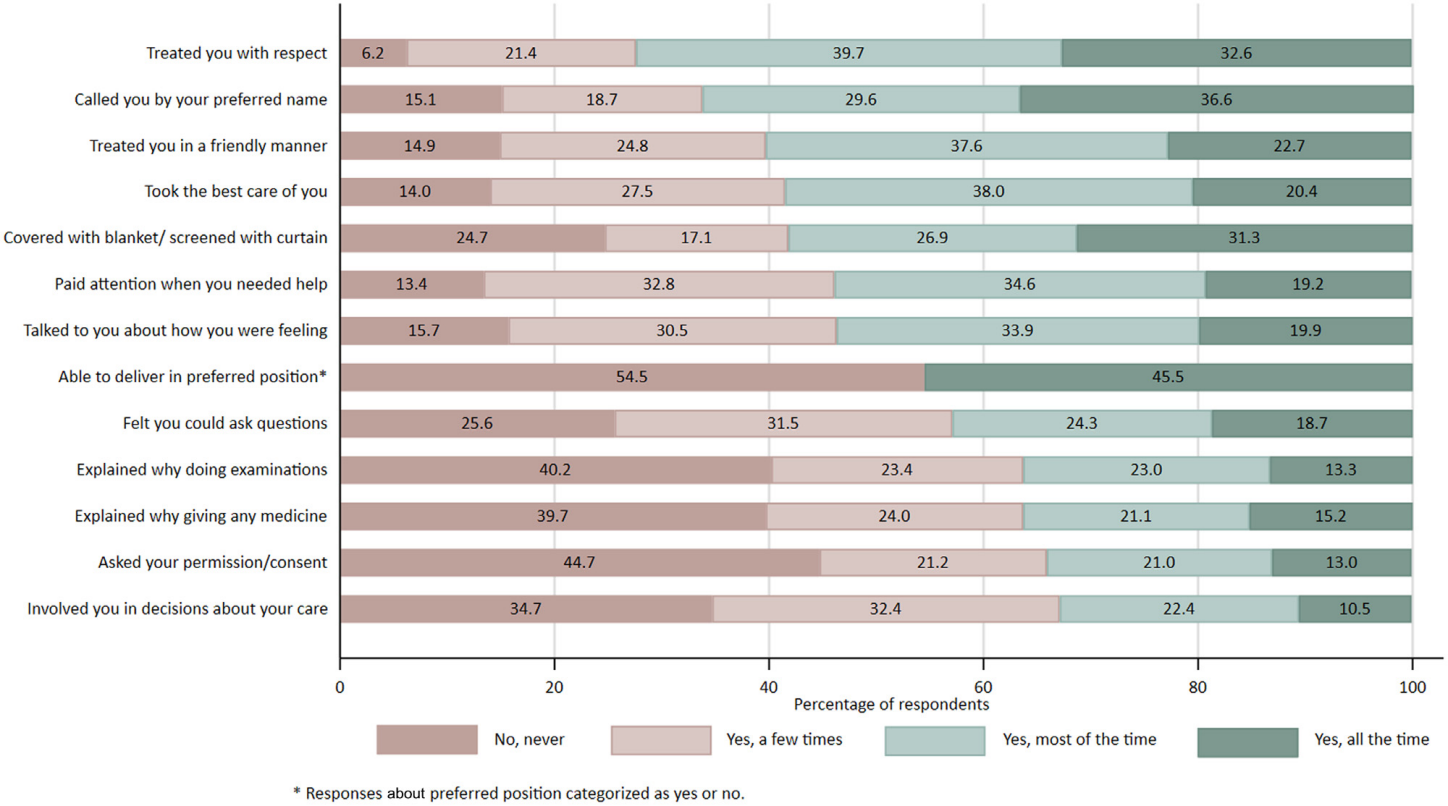
Responses to person-centered maternity care questions among women who delivered at a health facility

Overall, the weighted mean PCMC score in our sample was 19.07 (95% CI, 18.12–20.01) out of 39 points. Individual PCMC scores ranged from the minimum of zero to the maximum of 39 (Figure 1). Responses to items related to respect, preservation of dignity, trust, and supportive care were more favorable relative to other items. More than half of the respondents replied “yes, all the time” or “yes, most of the time” to the following items: treated you with respect, called you by your preferred name, treated you in a friendly manner, took the best care of you, covered you with blanket or screened with a curtain, paid attention when you needed help, and talked to you about how you are feeling (Figure 2, appendix p.6). Conversely, responses to items related to effective communication and supporting women's autonomy to make informed choices about her care were relatively lower. More than half of the respondents replied “no, never” or “yes, a few times” to the following items: able to be in the position that you preferred during delivery, felt you could ask any questions, examinations and procedures were explained, medications were explained, asked your permission or consent before performing examinations, and involved you in decisions about your care.

Stierman. Person-centered maternity care in Ethiopia. Am J Obstet Gynecol Glob Rep 2022.

Stierman. Person-centered maternity care in Ethiopia. Am J Obstet Gynecol Glob Rep 2022.

### Individual and community determinants of person-centered maternity care

Except for marital status, all individual and community characteristics showed a significant association with PCMC in unadjusted analyses (Table 2) (all *P* values <.05). However, the differences in unadjusted mean scores were relatively small (ie, <4 points on a 39-point scale) for most characteristics. The largest differences in mean PCMC score (ie, at least 4 points) were seen among women with different levels of wealth, education, modes of delivery (cesarean vs vaginal delivery), cadres of birth attendant (doctor, nurse, or midwife vs unknown provider type), place of delivery (private vs government facility), and region.

**Table 2:** Mean differences in person-centered maternity care scores by individual and community characteristics

Characteristics	Unadjusted		Adjusted^a^	
	Mean difference (95% CI)	*P* value	Mean difference (95% CI)	*P* value
**Individual characteristics**				
Age group (reference, 25–29 y)				
<20 y	−3.10 (−4.82 to −1.38)	<.001	−1.69 (−3.36 to −0.03)	.05
20–24 y	0.02 (−1.52 to 1.55)	.98	0.62 (−0.74 to 1.98)	.37
30–34 y	0.05 (−1.60 to 1.70)	.95	1.04 (−0.44 to 2.51)	.17
≥35 y	−1.34 (−2.93 to 0.25)	.10	0.42 (−1.08 to 1.91)	.58
Not currently married (reference, married)	−0.23 (−2.17 to 1.72)	.82	−0.63 (−2.56 to 1.30)	.52
Religion (reference, Orthodox)^b^				
Protestant	−2.79 (−4.59 to −0.99)	<.01	0.12 (−1.75 to 1.99)	.90
Muslim	−2.16 (−4.45 to 0.14)	.07	0.24 (−1.84 to 2.31)	.82
Wealth quintile (reference, middle)				
Lowest	−1.89 (−3.70 to −0·07)	.04	−1.18 (−2.92 to 0.57)	.19
Lower	−0.60 (−2.35 to 1.16)	.50	−0.26 (−2.03 to 1.50)	.77
Higher	1.90 (0.14–3.67)	.04	1.35 (−0.52 to 3.22)	.16
Highest	2.92 (1.01–4.84)	<.01	1.02 (−1.80 to 3.73)	.49
Education (reference, primary)				
Never attended	−1.38 (−2.69 to −0.07)	.04	−1.45 (−2.85 to −0.06)	.04
Secondary	2.01 (0.66–3.35)	<.01	0.25 (−0.90 to 1.39)	.67
Higher	3.99 (1.90–6.08)	<.001	1.14 (−0.90 to 3.18)	.27
Had at least 4 antenatal care visits or contacts^c^ (reference, no)	3.28 (2.01–4.56)	<.001	1.60 (0.58–2.62)	<.01
Cesarean delivery (reference, no)	4.70 (2.44–6.97)	<.001	3.57 (1.46–5.69)	<.01
Any complications^d^ (reference, none)	−1.24 (−2.42 to −0.05)	.04	−1.21 (−2.21 to −0.22)	.02
Stillbirth^e^ (reference, live birth)	−3.66 (−7.16 to −0.16)	.04	−2.52 (−6.93 to 1.90)	.26
Allowed family and friends during labor (reference, no)	2.09 (0.78–3.40)	<.01	1.55 (0.47–2.64)	<.01
Place of delivery^f^ (reference, government health center)				
Government hospital	−0.15 (−1.83 to 1.53)	.86	−0.77 (−2.36 to 0.81)	.34
Private facility	9.37 (6.61–12.12)	<.001	6.60 (3.83–9.38)	<.001
Provider attending delivery (reference, nurse or midwife)				
Doctor	0.91 (−0.90 to 2.72)	.32	−0.19 (−1.97 to 1.58)	.83
Other or not able to distinguish	−3.55 (−5.19 to −1.91)	<.001	−2.59 (−4.10 to −1.07)	<.01
**Community characteristics**				
Rural (reference, urban)	−2.56 (−4.39 to −0.74)	<.01	−0.27 (−2.14 to 1.60)	.77
Region (reference, Oromia)^g^				
Tigray	4.50 (2.08–6.91)	<.001	3.58 (1.16–6.00)	<.01
Amhara	2.74 (0.00–5.47)	.05	3.08 (0.39–5.77)	.03
SNNPR^h^	−1.88 (−4.27 to 0.51)	.12	−1.60 (−3.73 to 0.53)	.14
Addis Ababa	2.52 (−0.11 to 5.15)	.06	−0.12 (−3.04 to 2.81)	.94
Poor households in community,^i^ per 10% change	−0.37 (−0.57 to −0.17)	<.001	−0.26 (−0.66 to 0.13)	.19
Women in community with secondary education, per 10% change	0.78 (0.46–1.10)	<.001	0.47 (0.04–0.91)	.03
Community norms about facility delivery,^j^ per unit change	2.60 (0.91–4.28)	<.01	0.85 (−0.68 to 2.39)	.27

Estimates weighted to account for complex survey design and adjusted for variability between imputations.

CI, confidence interval. SNNPR, Southern Nations, Nationalities, and Peoples' Region.

a Adjusted for age group, marital status, religion, education, ≥4 antenatal care visits or contacts, cesarean delivery, complications, stillbirth, whether family and friends were allowed during labor, place of delivery, provider, urban or rural location, region, and community norms about facility delivery, with the following exceptions: because of multicollinearity, multivariable models did not control for wealth quintile, percentage of women in community with a secondary (or higher) education, and percentage of households in community that were poor; in addition, education was not controlled for in the multivariable model for wealth nor the model for percentage of women in community that had a secondary education

b Mean difference for women who identified as practicing another religion or being a non-believer are not shown because of the small sample size

c Antenatal contacts or visits could have been with any healthcare provider or health extension worker in a facility, home, or other location

d Any complications during delivery or first 24 hours following delivery

e In case of multiple births, it was categorized as stillbirth if any of the births were stillbirths

f Mean difference for women who delivered in government health posts not shown because of the small sample size

g Mean difference for women residing in Afar are not shown because of the small sample size

h SNNPR was subsequently divided into 3 regions, namely SNNPR, Sidama, and South West Ethiopia People's Region (SWER). The results are representative of political boundaries in September 2019

i Categorized as poor if household is in bottom 3 wealth quintiles, namely lowest, lower, and middle

j Community norms measured on a scale with a minimum of zero (female respondents in community perceived that no people encourage facility delivery) and maximum of 3 (all female respondents in community perceived that most people encourage facility delivery).*Stierman. Person-centered maternity care in Ethiopia. Am J Obstet Gynecol Glob Rep 2022.*

After adjusting for other covariates, a significant association remained between PCMC and the following characteristics: age group, education, at least 4 antenatal care contacts or visits, whether family and friends were allowed during labor, place of delivery, type of provider attending the delivery, cesarean delivery, complications during delivery or the first 24 hours postpartum, region, and the percentage of women in the community with a secondary (or higher) education. The largest adjusted differences in mean PCMC scores were seen between places of delivery and regions. The sensitivity analysis found no statistically significant difference in the PCMC scores for women delivering before or after the declaration of a state of emergency in Ethiopia owing to the COVID-19 pandemic (appendix p.7).

### Variation in person-centered maternity care scores within and between communities

**Figure 3:**
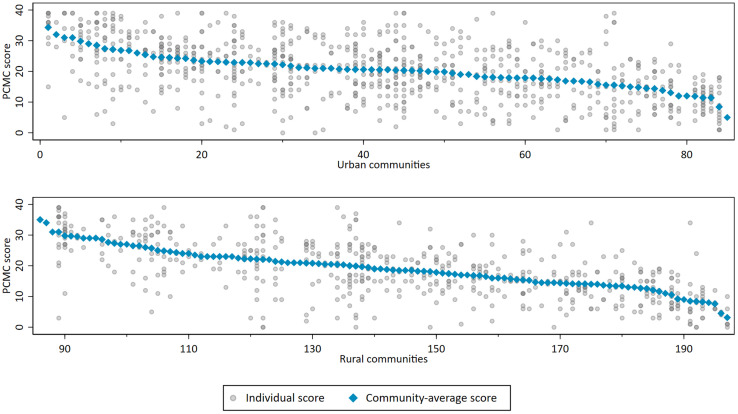
Variation in person-centered maternity care scores between communities and between individuals within communities Each unit on the x-axis corresponds to 1 community sorted, first, by urban or rural setting and, second, from highest to lowest community-average score. Scores for individuals residing in the same community are shown as separate *gray dots* that share a common x-value (ie, they are vertically aligned). *PCMC*, person-centered maternity care.

Variation in the PCMC scores between individuals residing within the same community (σ^2^ = 58.3) was nearly 3 times greater than variation between communities (τ^2^ =21.2) (Supplementary Material p.8). Within the same community, the range of individual PCMC scores varied as much as 39 points on the 39-point scale (Figure 3). Urban communities had a small but significantly greater deviation in the PCMC scores than rural communities (*P*=.02). After adjusting for community characteristics (rural or urban location, region, percentage of women with secondary education, percentage of poor households, community norms about facility delivery), variance between communities shrank by 30%. Adjusting for individual characteristics had more limited impact on reducing the variance between individuals (ie, σ^2^ shrank by 9%). After accounting for all measured variables, a substantial, unexplained variance remained between individuals’ maternity care experiences (ie, total variance shrank by 17% after accounting for all measured variables, with a remaining unexplained variance of σ^2^=53.0 and τ^2^=13.2).

Stierman. Person-centered maternity care in Ethiopia. Am J Obstet Gynecol Glob Rep 2022.

## Discussion

### Principal findings

Maternity care experiences varied widely among our sample of Ethiopian women delivering in health facilities, even among those residing within the same community. The mean PCMC score was 19.07 (95% CI, 18.12–20.01) out of 39 points, indicating the frequency of positive responses (ie, “yes, all the time” or “yes, most of the time”) was roughly equivalent to the frequency of negative responses (ie, “no, never” or “yes, a few times”). Women with greater wealth, more formal education, and nonadolescents reported significantly better PCMC.

### Results

PCMC varied significantly by patients’ sociodemographic characteristics. Individuals with no or limited formal education, lower wealth, and adolescents received relatively worse treatment than their counterparts. These findings are largely consistent with other studies, which have found lower wealth (or lower income) to be a key predictor of disrespect and abuse,^26^^,^^29^^,^^35^^,^^36^^,^^45^ and a multicountry study that found young age (15–19 years) to be the primary predictor of mistreatment.^3^ The relationship between education and respectful, person-centered care is less clear; some studies, including ours, have found that women with less formal education are more likely to report mistreatment,^34^^,^^45^ whereas other studies have found the opposite.^30^^,^^31^^,^^33^

Patients who were allowed to have family and friends with them during labor reported significantly higher PCMC, consistent with other studies that have found that the presence of a birth companion improved respectful care.^29^^,^^30^^,^^32^^,^^39^^,^^50^^,^^51^ Characteristics of the childbirth experience also influenced PCMC. Patients who experienced complications during delivery or within 24 hours postpartum reported relatively lower PCMC. Conversely, patients who had a cesarean delivery had higher PCMC scores than those who had vaginal deliveries; this includes both elective and medically indicated cesarean deliveries, making it challenging to interpret this association. Nevertheless, it is encouraging that women who underwent more complex procedures were more likely to report elements of person-centered care, such as being provided explanations, being asked for their consent, and having attention paid to them when they needed help. Of concern, one-third of patients were unable to distinguish the type of skilled attendant assisting their delivery, and these patients reported lower PCMC scores than those who identified their attendant as a doctor or a nurse or midwife. Patients’ inability to identify their provider's qualifications likely indicates poor communication and rapport, and it could be an indication that the provider was unknown to the patient before delivery (eg, the patient did not see this provider during antenatal care visits).

Similar to other studies in Ethiopia,^35^^,^^36^^,^^38^ we found that women who delivered in private facilities reported better treatment. However, this finding should be interpreted in the context. In Ethiopia, delivery in private facilities is relatively rare (3.2% in our sample), and nearly all those who delivered in private facilities were urban residents in the wealthiest quintile. Therefore, this finding may reflect more about the characteristics of this small subgroup of women than about the quality of care at private facilities relative to government facilities. Surprisingly, there were no significant differences in PCMC reported by those delivering in government hospitals when compared with government health centers; this is despite previous research documenting that hospitals in Ethiopia tend to have a greater availability of skilled health professionals, medicines, and other resources and systems to support patient safety and quality for childbirth care.^52^

At the community level, women residing in communities that were urban, with lower poverty levels, and higher levels of female educational attainment tended to report higher average PCMC scores in unadjusted analyses; after adjustment, higher levels of female educational attainment remained a significant predictor. However, there was substantial variation in PCMC among women residing within the same community. This is surprising, because we expected that community members would be more likely to share similar socioeconomic characteristics and cultural norms and to have similar access to childbirth care within a local health system comprised of the same facilities and their associated management, policies, and human resources. Instead, we found that community members often reported very different experiences. Variation is common even in rural communities where there may be only 1 nearby childbirthing facility with relatively few providers attending births. This may suggest that providers do not behave consistently; they may spend time explaining procedures and answering questions for one patient but not for the next or they may be more pleasant in the morning and less so in the afternoon. It could also point to the unique nature of interpersonal interactions between patients and providers, which are influenced by a myriad of factors, including patient expectations, providers’ beliefs and biases, workload, and characteristics of the delivery. Some of these factors (ie, demographic characteristics) are easily measurable, but many more are internal factors (ie, beliefs) that are difficult to measure and compare objectively between individuals. This may explain why the characteristics measured in our study, although significantly associated with PCMC, explained only a small portion of the total variation observed between individuals.

### Clinical implications

Our study underscores that a lot of work remains to meet the WHO standards for quality maternal and newborn care in health facilities.^6^ Three of the 8 standards (standards 4, 5, and 6) focus on quality of care as experienced by women. Standard 4 emphasizes the importance of effective communication, but we found that nonconsented care and poor communication practices are widespread. Similar to other studies in Ethiopia, we found that more than half of maternity patients reported that they were not usually asked for their consent before medical examinations or procedures,^11^^,^^26^, ^27^, ^28^, ^29^^,^^31^^,^^33^^,^^34^^,^^53^ and most usually felt that they could not ask questions.^28^^,^^33^^,^^53^^,^^54^ Standard 5 concerns respect and preservation of dignity. Patients responded more favorably to questions about being treated with respect and in a friendly manner, but physical privacy was not consistently protected. Standard 6 addresses emotional support that is sensitive to the patient's needs and strengthens their capabilities. We found that providers usually did not involve patients in decisions about their care. Similar to other studies, half the patients felt that they were not able to choose their preferred birthing position. One in 7 patients responded that providers never paid attention when they needed help.

To address these gaps, medical and nursing schools can help to promote better communication and person-centered care by emphasizing these standards and values in their curriculum and by providing students with opportunities to practice their interpersonal skills. Another promising strategy to improve PCMC is the use of birth companions or doulas. Although there have been relatively few studies on this topic in LMICs, this study and others have found that the presence of a support person was associated with more respectful, person-centered care,^32^^,^^55^ and interventions that promote companionship have shown potential to improve maternity care experiences.^56^

### Research implications

Our study found wide variation in the maternity care experiences of neighbors residing within the same community, signaling that there are highly unique characteristics and complex interactions between patients and providers that influence individual maternity care experiences. Further research is needed to understand the unexplained variation in maternity care experiences and the relative importance of unmeasured factors, such as provider stress and bias, and women's expectations of care.

### Strengths and limitations

This study assesses PCMC in a representative sample of Ethiopian women from 6 regions that together comprise 90% of the country's total population. In contrast, most previous studies on this subject were relatively small in size and were confined to a limited geographic area and a few health facilities, making it difficult to compare experiences across communities and facility types. The larger, representative sample and greater diversity of settings (eg, urban and rural, multiple regions) included in this study allowed for these comparisons.

Limitations of the study include its reliance on self-reported information. Although the PCMC scale was designed to minimize subjectivity by phrasing questions and response options in a manner designed to elicit factual descriptions, self-reported data inherently introduces a level of subjectivity.^57^ For example, the meaning one woman gives to being treated “with respect” or “in a friendly manner” could be quite different from another woman's interpretation. Moreover, poor treatment may be normalized in settings where the population has learned to expect such behavior from healthcare providers. Another limitation was data availability; we were not able to investigate all the provider, facility, and other characteristics that we hypothesized may influence PCMC because the survey collected limited information on these variables.

### Conclusion

In 2015, the government of Ethiopia launched the Health Sector Transformation Plan (HSTP) with a strategic focus on improving equity and quality of healthcare and on building a caring, respectful, and compassionate health workforce.^58^ The government recommitted to these goals in the HSTP-II, specifying priorities to promote ethics and professionalism in preservice and in-service education and to create an enabling work environment that fosters motivated, competent, and compassionate care.^59^ Our study provides information to policy makers, administrators, and providers on the gaps in meeting the WHO standards for quality maternity care. In particular, our findings highlight the need to reinforce effective communication practices and support women's autonomy during childbirth. Our findings also call attention to the disparities in care between sociodemographic groups in that women who are poor, have less formal education, and adolescents reported relatively worse treatment than others. Finally, our study finds widely varying maternity care experiences; even individuals residing within the same community reported large differences in how they were treated by healthcare providers. Many factors can influence why one interaction may be positive and another negative. Our study identified several factors associated with person-centered care, but a lot of the variation remains unexplained. This suggest that some of the reasons why providers fail to consistently deliver person-centered care could be because of internal (ie, stress, bias) and external factors (ie, crowded wards) that are difficult to measure.

## Appendix Supplementary materials

Image, application 1

## References

[1] United Nations Department of Economic and Social Affairs Population Division. Revision of world population prospects 2019. 2019. Available at: https://population.un.org/wpp/. Accessed October 6, 2020.

[2] United Nations Children's Fund. Data: delivery care. 2020. Available at:https://data.unicef.org/topic/maternal-health/delivery-care/. Accessed October 6, 2020.

[3] Bohren MA, Mehrtash H, Fawole B (2019). How women are treated during facility-based childbirth in four countries: a cross-sectional study with labour observations and community-based surveys. Lancet.

[4] World Health Organization (2014). https://apps.who.int/iris/handle/10665/134588.

[5] Tunçalp Ӧ, Were WM, MacLennan C (2015). Quality of care for pregnant women and newborns-the WHO vision. BJOG.

[6] World Health Organization (2016). https://apps.who.int/iris/handle/10665/249155.

[7] Kujawski S, Mbaruku G, Freedman LP, Ramsey K, Moyo W, Kruk ME (2015). Association between disrespect and abuse during childbirth and women's confidence in health facilities in Tanzania. Matern Child Health J.

[8] Bohren MA, Vogel JP, Hunter EC (2015). The mistreatment of women during childbirth in health facilities globally: a mixed-methods systematic review. PLoS Med.

[9] Bohren MA, Hunter EC, Munthe-Kaas HM, Souza JP, Vogel JP, Gülmezoglu AM. (2014). Facilitators and barriers to facility-based delivery in low- and middle-income countries: a qualitative evidence synthesis. Reprod Health.

[10] Miller S, Abalos E, Chamillard M (2016). Beyond too little, too late and too much, too soon: a pathway towards evidence-based, respectful maternity care worldwide. Lancet.

[11] Asefa A, Bekele D, Morgan A, Kermode M. (2018). Service providers’ experiences of disrespectful and abusive behavior towards women during facility based childbirth in Addis Ababa. Ethiopia. Reprod Health.

[12] Gebremichael MW, Worku A, Medhanyie AA, Edin K, Berhane Y. (2018). Women suffer more from disrespectful and abusive care than from the labour pain itself: a qualitative study from women's perspective. BMC Pregnancy Childbirth.

[13] Wassihun B, Zeleke S. (2018). Compassionate and respectful maternity care during facility based child birth and women's intent to use maternity service in Bahir Dar, Ethiopia. BMC Pregnancy Childbirth.

[14] Mengesha MB, Desta AG, Maeruf H, Hidru HD. (2020). Disrespect and abuse during childbirth in Ethiopia: a systematic review. BioMed Res Int.

[15] Topp SM, Chipukuma JM. (2016). A qualitative study of the role of workplace and interpersonal trust in shaping service quality and responsiveness in Zambian primary health centres. Health Policy Plan.

[16] Asefa A, McPake B, Langer A, Bohren MA, Morgan A. (2020). Imagining maternity care as a complex adaptive system: understanding health system constraints to the promotion of respectful maternity care. Sex Reprod Health Matters.

[17] Asefa A, Morgan A, Gebremedhin S (2020). Mitigating the mistreatment of childbearing women: evaluation of respectful maternity care intervention in Ethiopian hospitals. BMJ Open.

[18] Molla MM, Betemariam M, Fesseha W, Karim N (2017). Disrespect and abuse during pregnancy, labour and childbirth: a qualitative study from four primary healthcare centres of Amhara and Southern Nations Nationalities and People's Regional States. Ethiopia. Ethiop J Health Dev.

[19] Burrowes S, Holcombe SJ, Jara D, Carter D, Smith K. (2017). Midwives’ and patients’ perspectives on disrespect and abuse during labor and delivery care in Ethiopia: a qualitative study. BMC Pregnancy Childbirth.

[20] Engl E, Kretschmer S, Jain M (2019). Categorizing and assessing comprehensive drivers of provider behavior for optimizing quality of health care. PLoS One.

[21] Afulani PA, Kelly AM, Buback L, Asunka J, Kirumbi L, Lyndon A (2020). Providers’ perceptions of disrespect and abuse during childbirth: a mixed-methods study in Kenya. Health Policy Plan.

[22] Mdoe P, Mills TA, Chasweka R (2021). Lay and healthcare providers’ experiences to inform future of respectful maternal and newborn care in Tanzania and Malawi: an appreciative inquiry. BMJ Open.

[23] Downe S, Lawrie TA, Finlayson K, Oladapo OT. (2018). Effectiveness of respectful care policies for women using routine intrapartum services: a systematic review. Reprod Health.

[24] Afulani PA, Ogolla BA, Oboke EN (2021). Understanding disparities in person-centred maternity care: the potential role of provider implicit and explicit bias. Health Policy Plan.

[25] Afulani PA, Buback L, Kelly AM, Kirumbi L, Cohen CR, Lyndon A (2020). Providers’ perceptions of communication and women's autonomy during childbirth: a mixed methods study in Kenya. Reprod Health.

[26] Adinew YM, Hall H, Marshall A, Kelly J. (2021). Disrespect and abuse during facility-based childbirth in central Ethiopia. Glob Health Action.

[27] Banks KP, Karim AM, Ratcliffe HL, Betemariam W, Langer A. (2018). Jeopardizing quality at the frontline of healthcare: prevalence and risk factors for disrespect and abuse during facility-based childbirth in Ethiopia. Health Policy Plan.

[28] Bekele W, Bayou NB, Garedew MG. (2020). Magnitude of disrespectful and abusive care among women during facility-based childbirth in Shambu town, Horro Guduru Wollega zone. Ethiopia. Midwifery.

[29] Tekle Bobo F, Kebebe Kasaye H, Etana B, Woldie M, Feyissa TR (2019). Disrespect and abuse during childbirth in Western Ethiopia: should women continue to tolerate?. PLoS One.

[30] Gebremichael MW, Worku A, Medhanyie AA, Berhane Y. (2018). Mothers’ experience of disrespect and abuse during maternity care in northern Ethiopia. Glob Health Action.

[31] Mihret MS. (2019). Obstetric violence and its associated factors among postnatal women in a Specialized Comprehensive Hospital, Amhara Region, Northwest Ethiopia. BMC Res Notes.

[32] Sheferaw ED, Bazant E, Gibson H (2017). Respectful maternity care in Ethiopian public health facilities. Reprod Health.

[33] Siraj A, Teka W, Hebo H. (2019). Prevalence of disrespect and abuse during facility based child birth and associated factors, Jimma University Medical Center, Southwest Ethiopia. BMC Pregnancy Childbirth.

[34] Ukke GG, Gurara MK, Boynito WG. (2019). Disrespect and abuse of women during childbirth in public health facilities in Arba Minch town, south Ethiopia – a cross-sectional study. PLoS One.

[35] Wassihun B, Deribe L, Worede N, Gultie T. (2018). Prevalence of disrespect and abuse of women during child birth and associated factors in Bahir Dar town, Ethiopia. Epidemiol Health.

[36] Dagnaw FT, Tiruneh SA, Azanaw MM, Desale AT, Engdaw MT. (2020). Determinants of person-centered maternity care at the selected health facilities of Dessie town, Northeastern, Ethiopia: community-based cross-sectional study. BMC Pregnancy Childbirth.

[37] Sheferaw ED, Kim YM, van den Akker T, Stekelenburg J. (2019). Mistreatment of women in public health facilities of Ethiopia. Reprod Health.

[38] Bante A, Teji K, Seyoum B, Mersha A. (2020). Respectful maternity care and associated factors among women who delivered at Harar hospitals, eastern Ethiopia: a cross-sectional study. BMC Pregnancy Childbirth.

[39] Maldie M, Egata G, Chanie MG (2021). Magnitude and associated factors of disrespect and abusive care among laboring mothers at public health facilities in Borena District, South Wollo, Ethiopia. PLoS One.

[40] Bulto GA, Demissie DB, Tulu AS. (2020). Respectful maternity care during labor and childbirth and associated factors among women who gave birth at health institutions in the West Shewa zone, Oromia region, Central Ethiopia. BMC Pregnancy Childbirth.

[41] Afulani PA, Feeser K, Sudhinaraset M, Aborigo R, Montagu D, Chakraborty N. (2019). Toward the development of a short multi-country person-centered maternity care scale. Int J Gynaecol Obstet.

[42] Zimmerman L, Desta S, Yihdego M (2020). Protocol for PMA-Ethiopia: a new data source for cross-sectional and longitudinal data of reproductive, maternal, and newborn health. Gates Open Res.

[43] Afulani PA, Diamond-Smith N, Golub G, Sudhinaraset M. (2017). Development of a tool to measure person-centered maternity care in developing settings: validation in a rural and urban Kenyan population. Reprod Health.

[44] Afulani PA, Diamond-Smith N, Phillips B, Singhal S, Sudhinaraset M. (2018). Validation of the person-centered maternity care scale in India. Reprod Health.

[45] Afulani PA, Phillips B, Aborigo RA, Moyer CA. (2019). Person-centred maternity care in low-income and middle-income countries: analysis of data from Kenya, Ghana, and India. Lancet Glob Health.

[46] StataCorp (2017).

[47] Merlo J, Chaix B, Ohlsson H (2006). A brief conceptual tutorial of multilevel analysis in social epidemiology: using measures of clustering in multilevel logistic regression to investigate contextual phenomena. J Epidemiol Community Health.

[48] Patel SA, Sherman SG, Khatry SK (2016). An index of community-level socioeconomic composition for global health research. Soc Indic Res.

[49] Stephenson R, Tsui AO. (2003). Contextual influences on reproductive wellness in northern India. Am J Public Health.

[50] Balde MD, Nasiri K, Mehrtash H (2020). Labour companionship and women's experiences of mistreatment during childbirth: results from a multi-country community-based survey. BMJ Glob Health.

[51] Bohren MA, Berger BO, Munthe-Kaas H, Tunçalp Ö. (2019). Perceptions and experiences of labour companionship: a qualitative evidence synthesis. Cochrane Database Syst Rev.

[52] Stierman EK, Ahmed S, Shiferaw S, Zimmerman LA, Creanga AA. (2021). Measuring facility readiness to provide childbirth care: a comparison of indices using data from a health facility survey in Ethiopia. BMJ Glob Health.

[53] Asefa A, Bekele D. (2015). Status of respectful and non-abusive care during facility-based childbirth in a hospital and health centers in Addis Ababa, Ethiopia. Reprod Health.

[54] Rosen HE, Lynam PF, Carr C (2015). Direct observation of respectful maternity care in five countries: a cross-sectional study of health facilities in East and Southern Africa. BMC Pregnancy Childbirth.

[55] Bohren MA, Hofmeyr GJ, Sakala C, Fukuzawa RK, Cuthbert A. (2017). Continuous support for women during childbirth. Cochrane Database Syst Rev.

[56] Chaote P, Mwakatundu N, Dominico S (2021). Birth companionship in a government health system: a pilot study in Kigoma, Tanzania. BMC Pregnancy Childbirth.

[57] Larson E, Sharma J, Bohren MA, Tunçalp Ö. (2019). When the patient is the expert: measuring patient experience and satisfaction with care. Bull World Health Organ.

[58] Federal Democratic Republic of Ethiopia Ministry of Health (2015).

[59] Federal Democratic Republic of Ethiopia Ministry of Health (2021).

