# Supplementary material for: Understanding variation in person-centered maternity care: Results from a household survey of postpartum women in 6 regions of Ethiopia
Source: AJOG Glob Rep. 2022 Dec 5;3(1):100140. doi: 10.1016/j.xagr.2022.100140 (PMC9803839; doi:10.1016/j.xagr.2022.100140)
Supplement: Supplementary file 1 [file mmc1.docx]

**Supplementary Material**

**Table of Contents**

[Table 1. Short PCMC scale 2](#_Toc100420575)

[Table 2. Unweighted responses to PCMC questions among women who delivered at a health facility 4](#_Toc100420576)

[Table 3. Comparison of PCMC scores across study populations 5](#_Toc100420577)

[Table 4. Weighted responses to PCMC questions among women who delivered at a health facility 6](#_Toc100420578)

[Table 5. Mean difference in PCMC scores before vs. during COVID-19 pandemic† 7](#_Toc100420579)

[Table 6. Variance in PCMC scores between communities and between individuals within communities 8](#_Toc100420580)

Table 1. Short PCMC scale

| **No.** | **Theme** | **Question** | **Response** |
| --- | --- | --- | --- |
| 1 | Treated with respect | Did the doctors, nurses, or other staff at the facility treat you with respect? | No, never 0  Yes, a few times 1  Yes, most of the time 2  Yes, all of the time 3  Don’t remember -88  No response -99 |
| 2 | Friendly | Did the doctors, nurses, and other staff at the facility treat you in a friendly manner? | No, never 0  Yes, a few times 1  Yes, most of the time 2  Yes, all of the time 3  Don’t remember -88  No response -99 |
| 3 | Explain examinations, procedures | Did the doctors or nurses explain to you why they were doing examinations or procedures on you? | No, never 0  Yes, a few times 1  Yes, most of the time 2  Yes, all of the time 3  Don’t remember -88  No response -99 |
| 4 | Consent to procedures/exams | Did the doctors or nurses ask your permission/consent before doing procedures or examinations on you? | No, never 0  Yes, a few times 1  Yes, most of the time 2  Yes, all of the time 3  Don’t remember -88  No response -99 |
| 5 | Explain medicines | Did the doctors or nurses explain to you why they were giving you any medicine? | No, never 0  Yes, a few times 1  Yes, most of the time 2  Yes, all of the time 3  Did not get any medicine -77  Don’t remember -88  No response -99 |
| 6 | Able to ask questions | Did you feel you could ask the doctors, nurses or other staff at the facility any questions you had? | No, never 0  Yes, a few times 1  Yes, most of the time 2  Yes, all of the time 3  Don’t remember -88  No response -99 |
| 7 | Delivery position choice | During the delivery, do you feel like you were able to be in the position that you preferred? | No 0  Yes 1  Don’t remember -88  No response -99 |
| 8 | Involvement in care | Did you feel like the doctors or nurses at the facility involved you in decisions about your care? | No, never 0  Yes, a few times 1  Yes, most of the time 2  Yes, all of the time 3  Did not have to make any decisions -77  Don’t remember -88  No response -99 |
| 9 | Attention during stay | When you needed help, did you feel the doctors, nurses or other staff at the facility paid attention? | No, never 0  Yes, a few times 1  Yes, most of the time 2  Yes, all of the time 3  Don’t remember -88  No response -99 |
| 10 | Talk about feeling | Did the doctors or nurses at the facility talk to you about how you were feeling? | No, never 0  Yes, a few times 1  Yes, most of the time 2  Yes, all of the time 3  Don’t remember -88  No response -99 |
| 11 | Took best care | Did you feel the doctors, nurses or other staff at the facility took the best care of you? | No, never 0  Yes, a few times 1  Yes, most of the time 2  Yes, all of the time 3  Don’t remember -88  No response -99 |
| 12 | Visual privacy | During examinations in the labor room, were you covered up with a cloth or blanket or screened with a curtain so that you did not feel exposed? | No, never 0  Yes, a few times 1  Yes, most of the time 2  Yes, all of the time 3  Don’t remember -88  No response -99 |
| 13 | Called by name | Did the doctors, nurses, or other healthcare providers call you by your preferred name? | No, never 0  Yes, a few times 1  Yes, most of the time 2  Yes, all of the time 3  Don’t remember -88  No response -99 |

Table 2. Unweighted responses to PCMC questions among women who delivered at a health facility

|  | **No, never** | **Yes, a few times** | **Yes, most of the time** | **Yes, all the time** | **NR/ DR/ NA** |
| --- | --- | --- | --- | --- | --- |
|  | **n (%)** | **n (%)** | **n (%)** | **n (%)** | **n (%)** |
| Did the doctors, nurses, or other staff at the facility treat you with respect? | 102 (6·5%) | 346 (22·0%) | 623 (39·5%) | 501 (31·8%) | 4 (0·3%) |
| Did the doctors, nurses, and other staff at the facility treat you in a friendly manner? | 218 (13·8%) | 393 (24·9%) | 576 (36·6%) | 382 (24·2%) | 7 (0·4%) |
| Did the doctors or nurses explain to you why they were doing examinations or procedures on you? | 578 (36·7%) | 363 (23·0%) | 389 (24·7%) | 236 (15·0%) | 10 (0·6%) |
| Did the doctors or nurses ask your permission/consent before doing procedures or examinations on you? | 632 (40·1%) | 345 (21·9%) | 357 (22·7%) | 232 (14·7%) | 10 (0·6%) |
| Did the doctors or nurses explain to you why they were giving you any medicine? | 559 (35·5%) | 359 (22·8%) | 342 (21·7%) | 261 (16·6%) | 55 (3·5%) |
| Did you feel you could ask the doctors, nurses or other staff at the facility any questions you had? | 347 (22·0%) | 458 (29·1%) | 441 (28·0%) | 317 (20·1%) | 13 (0·8%) |
| During the delivery, do you feel like you were able to be in the position that you preferred? | 771 (48·9%) | NA | NA | 763 (48·4%) | 43 (2·7%) |
| Did you feel like the doctors or nurses at the facility involved you in decisions about your care? | 479 (30·4%) | 470 (29·8%) | 375 (23·8%) | 177 (11·2%) | 75 (4·8%) |
| When you needed help, did you feel the doctors, nurses or other staff at the facility paid attention? | 196 (12·4%) | 488 (31·0%) | 554 (35·2%) | 331 (21·0%) | 7 (0·4%) |
| Did the doctors or nurses at the facility talk to you about how you were feeling? | 217 (13·8%) | 461 (29·3%) | 547 (34·7%) | 344 (21·8%) | 7 (0·4%) |
| Did you feel the doctors, nurses or other staff at the facility took the best care of you? | 197 (12·5%) | 416 (26·4%) | 589 (37·4%) | 368 (23·4%) | 6 (0·4%) |
| During examinations in the labor room, were you covered up with a cloth or blanket or screened with a curtain so that you did not feel exposed? | 433 (27·5%) | 241 (15·3%) | 412 (26·1%) | 474 (30·1%) | 16 (1·0%) |
| Did the doctors, nurses, or other healthcare providers call you by your preferred name? | 225 (14·3%) | 271 (17·2%) | 459 (29·1%) | 595 (37·8%) | 26 (1·7%) |

Abbreviations: DR, don’t remember. NA, not applicable. NR, no response. PCMC, person-centered maternity care.

Note: Data reflects raw, unweighted values prior to imputation of missing values. Sample size is 1,576. This includes one observation with no response to any of the 13 questions; this observation was removed prior to subsequent analyses.

Table 3. Comparison of PCMC scores across study populations

|  |  | **Score Distribution** | | | |
| --- | --- | --- | --- | --- | --- |
|  | **Cronbach’s alpha** | **Mean** | **SD** | **Min** | **Max** |
| Ethiopia | 0·8932 | 19·86 | 9·26 | 0 | 39 |
| Kenya | 0·8216 | 24·16 | 8·16 | 2 | 39 |
| Ghana | 0·7644 | 15·64 | 4·57 | 6 | 29 |
| India | 0·8233 | 19·40 | 7·37 | 0 | 39 |

Abbreviations: Min, minimum. PCMC, person-centered maternity care. SD, standard deviation.

Note: Data reflect unweighted values to facilitate comparison across studies. The weighted mean PCMC score for Ethiopia was 19·07 (SD=19·01).

Table 4. Weighted responses to PCMC questions among women who delivered at a health facility

|  | **No, never** | | **Yes, a few times** | | **Yes, most of the time** | | **Yes, all the time** | |
| --- | --- | --- | --- | --- | --- | --- | --- | --- |
|  | **Weighted n** | **Weighted % (95% CI)** | **Weighted n** | **Weighted % (95% CI)** | **Weighted n** | **Weighted % (95% CI)** | **Weighted n** | **Weighted % (95% CI)** |
| Did the doctors, nurses, or other staff at the facility treat you with respect? | 98 | 6·2 (4·5-7·9) | 337 | 21·4 (17·8-25·0) | 626 | 39·7 (35·7-43·8) | 514 | 32·6 (28·4-36·8) |
| Did the doctors, nurses, and other staff at the facility treat you in a friendly manner? | 235 | 14·9 (11·6-18·3) | 391 | 24·8 (21·4-28·2) | 591 | 37·6 (33·7-41·5) | 357 | 22·7 (18·9-26·4) |
| Did the doctors or nurses explain to you why they were doing examinations or procedures on you? | 633 | 40·2 (36·1-44·3) | 369 | 23·4 (20·3-26·6) | 363 | 23·0 (19·8-26·3) | 209 | 13·3 (10·2-16·4) |
| Did the doctors or nurses ask your permission/consent before doing procedures or examinations on you? | 704 | 44·7 (40·0-49·4) | 334 | 21·2 (18·3-24·2) | 331 | 21·0 (18·1-24·0) | 205 | 13·0 (9·8-16·2) |
| Did the doctors or nurses explain to you why they were giving you any medicine? | 626 | 39·7 (35·8-43·7) | 378 | 24·0 (20·8-27·2) | 332 | 21·1 (18·1-24·1) | 239 | 15·2 (11·9-18·5) |
| Did you feel you could ask the doctors, nurses or other staff at the facility any questions you had? | 402 | 25.6 (21.6, 29.5) | 496 | 31.5 (27.3, 35.7) | 383 | 24.3 (20.7, 27.9) | 295 | 18.7 (14.9, 22.6) |
| During the delivery, do you feel like you were able to be in the position that you preferred? | 859 | 54·5 (49·5-59·5) | NA | NA | NA | NA | 716 | 45·5 (40·5-50·5) |
| Did you feel like the doctors or nurses at the facility involved you in decisions about your care? | 546 | 34·7 (29·9-39·4) | 510 | 32·4 (28·3-36·5) | 353 | 22·4 (18·7-26·2) | 165 | 10·5 (7·7-13·3) |
| When you needed help, did you feel the doctors, nurses or other staff at the facility paid attention? | 211 | 13·4 (10·5-16·3) | 516 | 32·8 (28·1-37·4) | 546 | 34·6 (30·1-39·2) | 302 | 19·2 (15·3-23·1) |
| Did the doctors or nurses at the facility talk to you about how you were feeling? | 248 | 15·7 (12·7-18·8) | 481 | 30·5 (26·8-34·2) | 534 | 33·9 (29·8- 38·0) | 313 | 19·9 (16·1- 23·6) |
| Did you feel the doctors, nurses or other staff at the facility took the best care of you? | 221 | 14·0 (10·8- 17·3) | 433 | 27·5 (24·1-30·9) | 599 | 38·0 (34·0-42·1) | 322 | 20·4 (16·9-23·9) |
| During examinations in the labor room, were you covered up with a cloth or blanket or screened with a curtain so that you did not feel exposed? | 389 | 24·7 (20·5-28·8) | 269 | 17·1 (13·9-20·2) | 424 | 26·9 (23·4-30·4) | 494 | 31·3 (27·1-35·6) |
| Did the doctors, nurses, or other healthcare providers call you by your preferred name? | 239 | 15·1 (11·9-18·4) | 294 | 18·7 (14·9-22·4) | 466 | 29·6 (25·5-33·7) | 577 | 36·6 (31·5-41·7) |

Abbreviations: CI, confidence interval. NA, not applicable. PCMC, person-centered maternity care.

Note: Estimates weighted to account for complex survey design and adjusted for variability between imputations. Rows may not sum to 1,575 due to rounding.

Table 5. Mean difference in PCMC scores before vs. during COVID-19 pandemic

|  | **Unadjusted** | | **Adjusted†** | |
| --- | --- | --- | --- | --- |
|  | **Mean difference (95% CI)** | **p-value** | **Mean difference (95% CI)** | **p-value** |
| Before COVID-19 pandemic | Reference | NA | Reference | NA |
| During COVID-19 pandemic | -0·16 (-1·24 to 0·92) | 0·77 | -0·43 (-1·61 to 0·75) | 0·47 |

Abbreviations: CI, confidence interval. NA, not applicable. PCMC, person-centered maternity care.

Note: Estimates weighted to account for complex survey design and adjusted for variability between imputations. A woman delivered before the COVID-19 pandemic if delivery date is prior to April 8, 2020 (weighted n=1,170; 74·3%). A woman delivered during the COVID-19 pandemic if delivery date is April 8, 2020 or later (weighted n=405; 25·7%). April 8, 2020 is the date a state of emergency was declared in Ethiopia in response to the COVID-19 pandemic.

† Adjusted for age group, marital status, religion, wealth, education, 4+ antenatal care visits/contacts, cesarean delivery, complications, stillbirth, whether family and friends were allowed during labor, place of delivery, provider, urban/rural location, region, percentage of households in community that are poor, percentage of women in community that have a secondary education, and community norms about facility delivery.

Table 6. Variance in PCMC scores between communities and between individuals within communities

|  | **Empty model** | **Model adjusted for community characteristics+** | **Model adjusted for individual characteristics†** | **Model adjusted for individual and community characteristics‡** |
| --- | --- | --- | --- | --- |
| **Fixed effects** |  |  |  |  |
| Mean PCMC (intercept) | 18·9 | 17·4 | 19·2 | 17·0 |
| **Random effects** |  |  |  |  |
| Variance between communities τ^2^ | 21·2 | 14·7 | 15·5 | 13·2 |
| Variance between individuals within communities σ^2^ | 58·3 | 58·3 | 53·1 | 53·0 |
| Total variance τ^2^ + σ^2^ | 79·5 | 73·1 | 68·6 | 66·2 |
| Intracluster correlation ρ | 0·27 | 0·20 | 0·23 | 0·20 |

Abbreviations: PCMC, person-centered maternity care.

Notes: Coefficients and standard errors were adjusted for the complex survey design and for the variability between imputations using the ‘mi estimate: mixed’ commands in Stata version 15, with a with a random intercept for community and sampling weights (‘pweight’) to account for the EA selection probability.

+ Adjusted for rural or urban location, region, percentage of women in the community with a secondary (or higher) education, percentage of poor households in the community, and community norms about facility delivery.

† Adjusted for women’s age, education, wealth quintile, 4+ antenatal care visits/contacts, whether family and friends allowed during labor, place of delivery, type of provider attending the delivery, and characteristics of the delivery: birth outcome, cesarean delivery, and complications during delivery or the first 24 hours postpartum.

‡ Adjusted for above individual and community variables.
